# Detection of *Wuchereria bancrofti* infection in mosquitoes in areas co-endemic with *Brugia malayi* in Balasore district, Odisha, India

**DOI:** 10.1038/s41598-024-67188-2

**Published:** 2024-07-22

**Authors:** Philip Raj Abraham, Balasubramaniyan Ramalingam, Priyadarshini Mohapatra, Kaliannagounder Krishnamoorthy, Sugeerappa Laxmanappa Hoti, Ashwani Kumar

**Affiliations:** 1 ICMR-Vector Control Research Centre, Puducherry, India; 2 Saveetha Institute of Medical and Technical Sciences, Saveetha University, Thandalam, Kanchipuram, 602105 Tamil Nadu India

**Keywords:** *Brugia malayi*, *Culex quinquefasciatus*, Filariasis, Vector borne disease, *Wuchereria bancrofti*, Microbiology, Molecular biology, Diseases, Medical research

## Abstract

Lymphatic filariasis (LF) is a crippling and disfiguring parasitic condition. India accounts for 55% of the world’s LF burden. The filarial parasite *Wuchereria bancrofti* is known to cause 99.4% of the cases while, *Brugia malayi* accounts for 0.6% of the issue occurring mainly in some pockets of Odisha and Kerala states. The Balasore (Baleswar) district of Odisha has been a known focus of *B. malayi* transmission. We employed molecular xenomonitoring to detect filarial parasite DNA in vectors. In six selected villages, Gravid traps were used to collect *Culex* mosquitoes and hand catch method using aspirators was followed for collection of mansonioides. A total of 2903 mosquitoes comprising of *Cx. quinquefasciatus* (n = 2611; 89.94%), *Cx. tritaeniorhynchus* (n = 100; 3.44%), *Mansonia annuliferea* (n = 139; 4.78%) and *Mansonia uniformis* (n = 53; 1.82%) were collected from six endemic villages. The species wise mosquitoes were made into 118 pools, each with a maximum of 25 mosquitoes, dried and transported to the laboratory at VCRC, Puducherry. The mosquito pools were subjected to parasite DNA extraction, followed by Real-time PCR using LDR and HhaI probes to detect *W. bancrofti* and *B. malayi* infections, respectively. Seven pools (6.66%) of *Cx. quinquefasciatus,* showed infection with only *W. bancrofti* while none of the pools of other mosquito species showed infection with either *W. bancrofti* or *B. malayi.* Although the study area is endemic to *B. malayi*, none of the vectors of *B. malayi* was found with parasite infection. This study highlights the ongoing transmission of bancroftian filariasis in the study villages of Balasore district of Odisha and its implications for evaluating LF elimination programme.

## Introduction

The three primary nematode parasite species that cause lymphatic filariasis (LF), namely *Wuchereria bancrofti, Brugia malayi,* and *Brugia timori,* are responsible for the disease's transmission by mosquito species of the *Culex, Anopheles,* and *Mansonia*^1^. According to estimates by the World Health Organization (WHO), over 120 million people are already afflicted with the disease, and roughly 1.3 billion individuals pose a risk of infection^2^. The *W. bancrofti* is the most common and extensively dispersed parasite species and responsible for 90% of LF, in Africa, Southeast Asia, and the Western Pacific while, *B. malayi* is prevalent in Southeast Asia and *B. timori* is found only in Indonesia^3^ and Timor Leste^4^. In India, *W. bancrofti* is known to cause 99.4% of the cases while, *B. malayi* is responsible for 0.6% of the problem^5^. Both brugian and bancroftian infections are prevalent throughout southern parts of India, Odisha, Madhya Pradesh, Assam, and West Bengal^6^. Currently, 339 districts are endemic for LF in India^7^.

In India, filariasis elimination programme was launched in 2004 and expanded to cover all the then 256 endemic districts. The main strategy of the elimination programme is the annual Mass Drug Administration (MDA) with diethylcarbmazine and later albendazole in 2007. Currently the programme is covering 339 districts and stopped in 138 districts following the successful demonstration of interruption as evidenced by transmission assessment survey (TAS). Ivermectin is added to two drugs in 2019 and initially covered 5 districts and then expanded to 63 districts in 2023^5^. Now the programme is conducted in two phases, one in February and another in August (Ministry of Health and Family Welfare, 2022; https://pib.gov.in/PressReleasePage.aspx?PRID=2004769).

Odisha state is known to be endemic for both brugian and bancroftian filarial infections. In 1989, a new focus of *B. malayi* was detected in Chudamani, in Balasore (Baleswar) district of Orissa for the first time^8^. Recently, Balasore district has been reported as a focus of *B. malayi* transmission^9^. Similarly, high prevalence of *W. bancrofti* infection has been reported from five districts i.e., Bargarh, Balangir, Kandhamal, Kendujhar, and Sambalpur in western Odisha. These districts were earlier considered to be non-endemic^10^. Mass Drug Administration (MDA) programme was implemented in Odisha in 2004 to achieve the objective of LF elimination as a public health problem by 2027. The initial assessment of impact of the MDA programme depended on night blood smear which is cumbersome, time-consuming and lacks the desired sensitivity. For final assessment, the Transmission Assessment Survey (TAS) is carried out to verify absence of transmission. Filariasis Test strips (FTS) are recommended for TAS which is not only cost prohibitive, but also with short-expiry period. Hence, molecular xenomonitoring in vectors as an alternative tool to evaluate transmission disruption was taken into account^11–16^. Collection of *Mansonia* mosquitoes, presents notable challenge due to their distinct behavior, making the process of collecting and testing *Mansonia* mosquitoes a labor-intensive and resource-demanding technique. Alternatively, *Culex quinquefasciatus* mosquitoes can be used as proxy to check for parasite infection. In the current study we carried out molecular xenomonitoring of *W. bancrofti* and *B. malayi* in *Culex* and *Mansonia* spp., respectively collected from LF endemic villages of Balasore district, Odisha.

## Methods

### Study area

**Figure 1:**
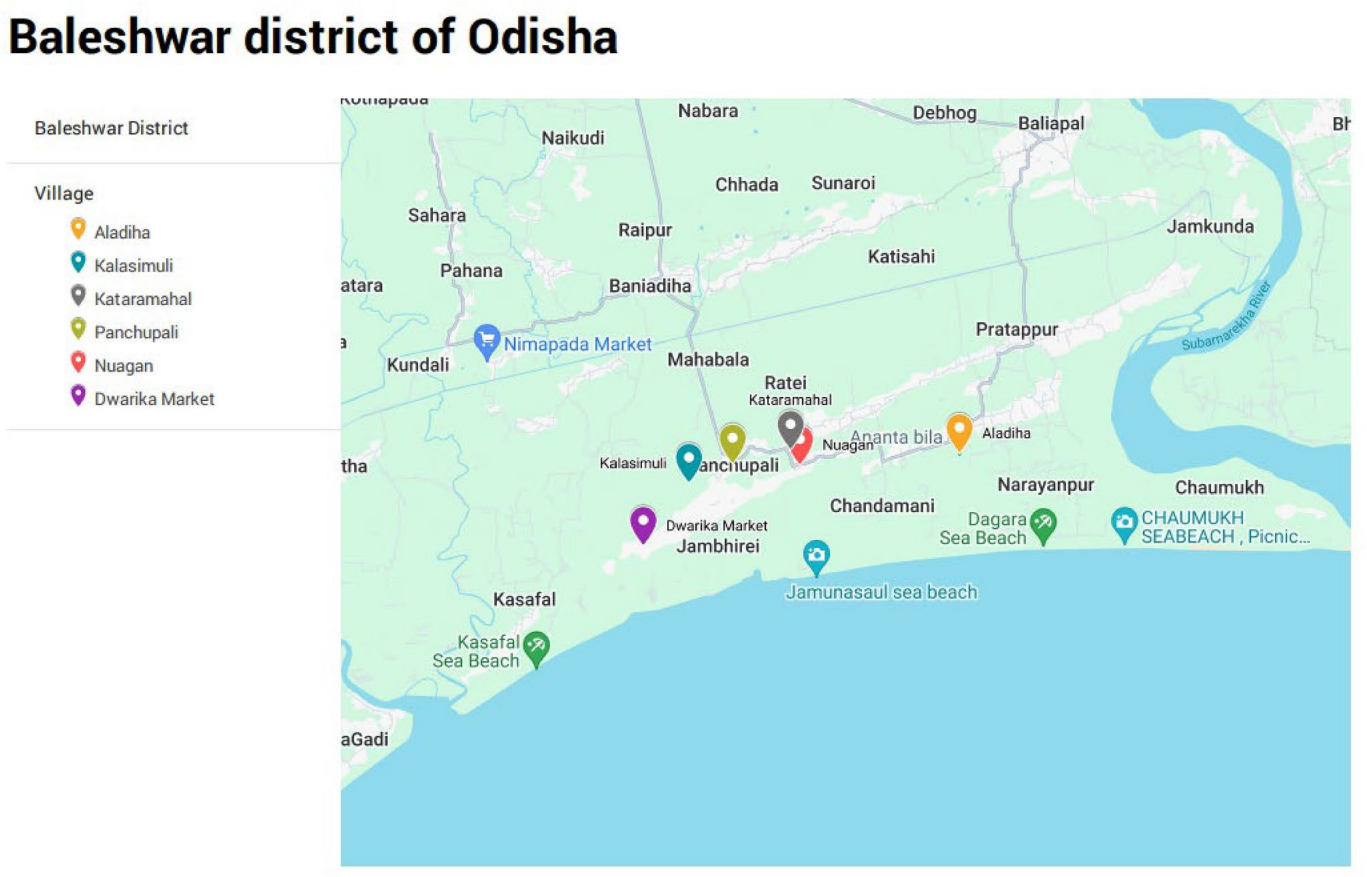
Map of study villages of Balasore district, Odisha. *Source*: Map generated with the Google Map.

**Figure 2:**
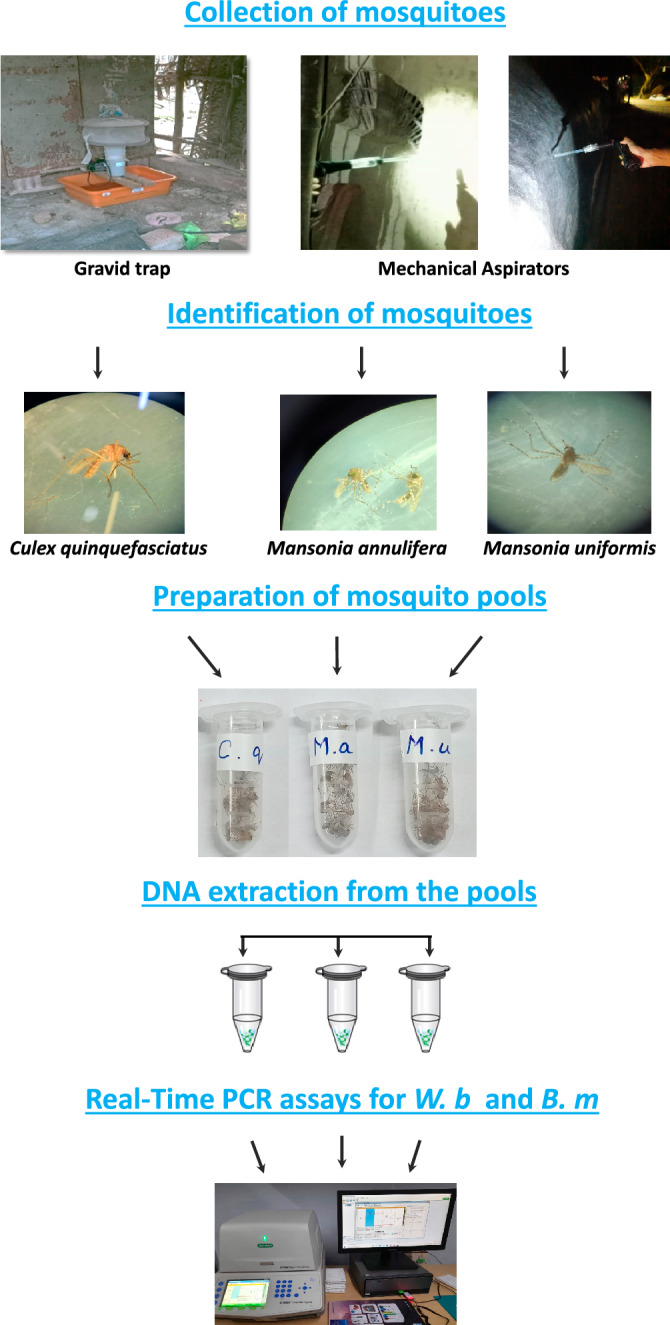
Outline of molecular xenomonitoring activities. Mosquitoes were collected from the study villages using the Gravid traps or mechanical aspirators. The mosquitoes were identified, pooled and transferred to the laboratory. Real-time PCR amplification was carried out using species-specific probes after extraction of DNA form the mosquito pools. *B.m* – *Brugia malayi; W.b* – *Wuchereria bancrofti.*

This research study was conducted from September, 2022 to April, 2023 in Balasore district, Odisha, India. Balasore, a coastal district of Odisha, is known to be the old focus of lymphatic filariasis. Both *Wuchereria bancrofti* and *Brugia malayi* parasites are prevalent in this district^9^. According to the endemicity of the district, six villages namely, Aladiha, Dwarika, Kalasimuli, Katramahal, Nuagaon and Panchupalli were selected for the study (Fig. 1). We followed standard protocol of molecular xenomonitoring to know the filarial infection in the mosquito vectors. These details are represented in Fig. 2.

### Mosquito collection

Mosquitoes were collected during the months of September and October 2022 following a standardized procedure of ICMR-Vector Control Research Centre (VCRC), viz. Gravid trap method of *Culex* mosquito collection^16^. In brief, mosquitoes were collected by modified CDC Gravid trap using hay infusion as oviposition attractant. The Gravid traps were placed near the selected houses around 6 PM. The traps were left overnight and the head of the family was informed about the survey. Adult mosquitoes trapped were collected, the following day by 6 AM and transferred to the field-laboratory at the Filariasis Control Unit of Balasore. The mosquitoes were carefully transferred to test tube using mechanical aspirator, identified using the key of Barraud^17^, pooled (25 mosquitoes/pool), dried and transferred to VCRC, Puducherry to check for filarial infection by RT-PCR assay. Since the gravid traps were less productive for the vectors of *B. malayi,* sweep-net collections were made for outdoor resting mosquitoes (06:00 AM to 07:00 AM) and hand catch collections using aspirators were made for indoor resting mosquitoes (07:00 AM to 09:00 AM).

### DNA extraction from mosquito pools

Tris–EDTA (TE) buffer-based boiling method was followed for DNA extraction from the dried mosquito pools^18^. Briefly, the working table surface was cleaned with 70% ethanol to avoid surface contamination. A 1X TE buffer (pH 8.0) was prepared from 10X stock solution (Medox Biotech India Pvt. Ltd.). From this, 100 µl buffer was added to the mosquito pools and homogenized in TissueLyser II (Qiagen) for 15 min at 25 frequency/sec. The homogenate was gently vortexed, boiled for 10 min and centrifuged. The supernatant containing the DNA sample was used for further processing.

### Real-time PCR assay

Real-Time quantitative PCR (qPCR) assay was carried out using specific primers and probes to check for the infection of *B. malayi* as well as *W. bancrofti* in mosquito pools, separately^19,20^. To detect the *B. malayi* infection, forward primer, HhaI- For- 5’GCGCATAAATTCATCAGC-3’, reverse primer, HhaI- Rev- 5’GCGCAAAACTTAATTACAAAAGC-3’ and TaqMan HhaI-probe- FAM-ACGTGAATTGTACCAGTGCTGGTCG-TAMRA were used. The PCR mixture contained 12.5 µl of TaqMan master mix, primers (1.0 µl each), probe (10 pM; 0.312 µl), and 1.0 µl of the DNA template and nuclease-free water (9.188 µl) in a final volume of 25 µl reaction mixture. For positive controls, the genomic DNA of *B. malayi* was used. The cycling conditions were 50 °C for 2 min, 95 °C for 10 min, followed by 40 cycles of 95 °C for 15s and 60^0^ C for 1 min.

To check for the *W. bancrofti* infection, DNA from the same pools of mosquitoes was subjected to qPCR assay using long DNA repeat (LDR) forward primer, LDR1-For-5’ATTTTGATCATCTGGGAACGTTAATA-3’, reverse primer, LDR2-Rev-5’CGACTGTCTAATCCATTCAGAGTGA-3’ and Probe-FAM-ATCTGCCCATAGAAATAAACTACGGTGGATCTG- TAMRA. The cycling parameters for qPCR remained same as mentioned for detection of *B. malayi* infection. For positive controls, the genomic DNA of *W. bancrofti* was used and for negative control, nuclease free water was used.

### Data analysis

A pool was considered ‘positive’ if the Cycle threshold (Ct) values of samples ranged from 1.0 to 39.0. Indeterminate pool (a mosquito pool was unable to attain the required level of fluorescence above 39) was repeated for confirming the pool as negative or positive^20^. The percentage of pool positivity was calculated by dividing the total number of pools where filarial parasite DNA is positive over total number of pools screened^16,21^.

## Results

### Distribution of mosquito species and composition of pools

A total of 2903 female mosquitoes were collected from six villages of Balasore district during the months of September and October 2022. Among the *Culex* spp., highest percentage of mosquitoes belonged to *Cx. quinquefasciatus* (89.94%), followed by *Cx. tritaeniorhynchus* (3.44%), but only 4.78% and 1.82% of the mosquitoes belonged to *Ma. annulifera* and *Ma. uniformis* respectively (Table 1). The *Cx. quinquefasciatus* mosquitoes were collected from all the six villages while, *Cx. tritaeniorhynchus* was collected only from Panchupalli village. Similarly, *Ma. annulifera* was collected from KalasimuIi and Panchupalli villages while *Ma. uniformis* was collected only from Kalasimuli. Highest number (1050; 36.16%) of mosquitoes were collected from Panchupalli village that included all the species of mosquito except *Ma. uniformis*. A total of 118 pools were prepared that comprised 105 pools of *Cx. quinquefasciatus* mosquitoes, followed by 4 pools of *Cx. tritaeniorhynchus*, 6 pools of *Ma. annulifera* and 3 pools of *Ma. uniformis*. These pools were subjected to qPCR for *W. bancrofti* and *B. malayi* infection.

**Table 1:** Village-wise details of mosquito collection.

S. No	Mosquito species	Aladiha	Dwarika	Kalasimuli	Katramahal	Nuagaon	Panchupalli	Total female
1	*Culex quinquefasciatus*	700	350	509	102	100	850	2611
2	*Culex tritaeniorhynchus*	–	–	–	–	–	100	100
3	*Mansonia annulifera*	–	–	39	–	–	100	139
4	*Mansonia uniformis*	–	–	53	–	–	–	53
Total		700	350	601	102	100	1050	2903

### Pool positivity in LF endemic villages

Initially, 9 pools of *Mansonia* spp. mosquitoes were subjected to *B. malayi* real-time qPCR. Since there was no infection found in these pools, 109 non-vector pools of *Culex* mosquito spp. were checked for *B. malayi* infection. None of the pools showed *B. malayi* infection. However, when they were tested for *W. bancrofti,* 7 out of 105 pools of *Cx. quinquefasciatus* showed positivity (6.7%) while, other non-vector mosquitoes didn’t show any infection **(**Table 2). Among the 7 positive pools, 4 pools from Panchupalli village followed by 2 positive pools from Aladiha and one pool from Kalasimuli.

**Table 2:** Mosquito pools showing positive pools for filarial infection.

Species of mosquitoes	No. of mosquitoes	No. of pools	No. of pools positivity for *B. malayi (Hha1 Probe)*	No. of Pools positivity for *W. bancrofti (LDR Probe)*
*Culex quinquefasciatus*	2611	105	0	7
*Culex tritaeniorhynchus*	100	4	0	0
*Mansonia annulifera*	139	6	0	0
*Mansonia uniformis*	53	3	0	0
Total	2903	118	0	7

## Discussion

Lymphatic filariasis is a debilitating parasitic illness that affects people all over tropical and subtropical areas of the world. With an intention of elimination of LF as a public health problem by 2020, the global program to eliminate lymphatic filariasis (GPELF) was launched in 2000, and has advanced remarkably. Balasore district of Odisha, India has been covered under annual MDA with DEC and albendazole since 2004 onwards. With over 65% coverage of MDA, the preTAS showed persistence of infection with Mf rate above 1%^5^. During a coverage and compliance survey in 2002, Balasore district has reported the highest coverage (78.1%) but low (54.4%) consumption^22^. Since 2004, there was no report of *B. malayi* in the annual Mf surveys from sentinel and random sites by the Lymphatic Filariasis Elimination Programme of Odisha. However, a recent study in 12 villages showed that Mf prevalence in 6230 individuals tested was 0.99%, with 5 villages having more than 1% threshold of Mf prevalence. Of the total infection, *B. malayi* infection was 98.4% and the remaining was *W. bancrofti* in Balasore district^9^. In addition, the data collected by ICMR-VCRC, Puducherry through Brugia Rapid Test kit and the night blood survey provides evidence of Brugian infection in the Balasore district (unpublished report). The present study reports for the first time the results of molecular xenomonitoring of filarial parasites in *Culex* and *Mansonia* mosquitoes.

Traditionally, Culex mosquitoes have been primarily associated with the transmission of *W. bancrofti*, while *Mansonia* mosquitoes to *B*. *malayi* transmission. Initially, we tried to collect mansonioides to find the *B. malayi* infection. However, we could not collect sufficient number of mansonioides due to low prevalence, peculiar oviposition behavior, less attractive collection method, and also less abundance of *Mansonia* spp. during the study period, we collected *Culex* spp. with assumption that Mf are ingested while feeding on human by these anthropophilic mosquitoes. We collected high density of *Culex* mosquitoes during the vector collection period. The mosquitoes were subjected to detect *W. bancrofti* and *B. malayi* infection in known *Culex* spp, as non-vector mosquitoes also play role in the transmission of filariasis. However, there was no pool of mosquitoes with *B. malayi* infection in the non-vector as well as vector mosquitoes. Instead, when we looked for *W. bancrofti* infection in its principal vector *Cx. quinquefasciatus* positive pools (6.7%) were observed though the villages selected for the study are reported to be endemic for *B. malayi* infection. Similar studies have demonstrated high percentage of positive pools for *W. bancrofti* infection in *Cx. quinquefasciatus*. McPherson et al.^23^ reported 18% (86/475) pools positive for *W. bancrofti* DNA by PCR in primary sampling units of Samoa. While, in American Samoa, 5/585 pools were positive out of 4,413 *Cx. quinquefasciatus* mosquitoes tested^24^. In Tanzania, molecular xenomonitoring was carried out to assess the infection status in mosquito vectors after the MDA. Masasi district showed 9% pools (33/365 pools) and Rufiji district showed 0.45% pools (5/1092 pools; total 5460 mosquitoes) positive in mosquito vectors tested after six and twelve rounds of MDA^25,26^. Srilankan studies have showed 14.7% pools (92/625) positive in coastal and 1.4% (8/583) pools positive in inland evaluation units^13^. In another study, filarial DNA rate in mosquitoes collected in Matotagama from February 2013 to June 2014 reported positive pools ranging from 0 to 23.4%^27^. Studies have also reported no positivity in pools of *Cx. quinquefasciatus* mosquitoes. In Gaibandha and Panchagarh districts of Bangladesh, out of 594 pools (total 10,021 *Cx. quinquefasciatus* mosquitoes) none of the pool was tested positive^28^. Similarly, *Culex* and *Mansonia* mosquitoes (1801 and 1823 mosquitoes respectively) were negative for *W. bancrofti* infection in four districts of Ghana^29^. In Indian settings, molecular xenomonitoring survey showed 11 (3.1%) of the 358 pools (8850 *Cx*. *quinquefasciatus*) positive in Cuddalore district of Tamilnadu^15^. Recently, molecular xenomonitoring was carried out to check the infection in vector mosquitoes during validation of mini-TAS in two non-MDA districts of Odisha. Out of 7990 *Cx*. *quinquefasciatus* mosquitoes tested, 3.3% pools (10/300 pools) found to be positive^30^. However, in this study we observed (6.7%) pools positive for *W. bancrofti* infection.

Detection of filarial infection in the *Culex* mosquitoes offers several advantages compared to the Filariasis Test Strip (FTS) kits. Firstly, the FTS kits are expensive and have a short expiry date, which can limit their availability and practicality for large-scale studies. Furthermore, the invasive procedure of the FTS kits limits its applicability for routine surveillance and monitoring purposes. In contrast, the non-invasive collection of *Culex* mosquitoes offers a more practical and efficient approach through molecular xenomonitoring. It plays a crucial role in elucidating the transmission patterns of LF. By detecting parasite DNA in mosquito vectors, this technique provides valuable information on the presence and prevalence of infection in specific vector species, helping to identify high-risk areas and inform targeted control measures. Molecular xenomonitoring does not appear to be more practical as it requires laboratory and skilled technicians. However, this can be solved at field level by establishing regional laboratories for processing the mosquitoes for molecular assay and training grass root level laboratory technicians in sampling mosquitoes, identification and storing. It should be possible as the collection is seasonal and the stored mosquitoes can be processed by pooling samples from different locations. Njenga et al.^31^ conducted integrated epidemiological surveys using standard parasitological and entomological methods in Kenya and provided useful information on co-endemic parasitic diseases. The coexistence of *B. malayi*^9^ with *W. bancrofti* (current study) was evident during MDA. However, the present study showed only the presence of *W. bancrofti* infection in highly anthropophilic *Culex quinquefasciatus* mosquitoes. This suggests possible disappearance of *B. malayi* infection from the area as it is more susceptible to DEC. Studies have established the fact that *B. malayi* parasite is more susceptible to DEC, compared to *W. bancrofti*^32^. The parasite species has probably either disappeared or reduced to undetectable levels due to treatment and by the restricted distribution of host plants (*Pistia striatus*) due to change in the ecology and human activities. Our study has not detected any *B. malayi* parasite in mosquitoes in the study area, suggesting low prevalence of *B. malayi* infection. However, more studies with adequate sample size are required to confirm the absence of *B. malayi* in *Mansonia* mosquitoes and its transmission.

We observed that even full-fed female *Culex* mosquitoes do not show any evidence of *B. malayi* parasite infection. This shows that the same geographical area, molecular xenomonitoring provides valuable insights into the transmission dynamics of LF due to two parasites. This observation suggests that individuals in the area may be at risk of acquiring multiple filarial infections, potentially leading to more severe clinical outcomes and challenges in treatment. Understanding the prevalence and distribution of both the species of parasites is crucial for tailoring interventions and optimizing treatment approaches in areas with multiple parasite species that circulate in a community. Molecular xenomonitoring is one such tool which can be employed to understand the transmission dynamics of filarial parasites when they co-exist.

## Conclusion

This study provides valuable insights to the ongoing transmission of *W. bancrofti* and highlights the importance of molecular xenomonitoring using *Culex* mosquitoes for understanding LF transmission dynamics in Balasore district of Odisha. This information is crucial for designing effective control and surveillance strategies for elimination of lymphatic filariasis in this region.

## Data Availability

The data supporting the conclusions of this article are included within the article. The datasets analysed during the present study are available from the corresponding author (PRA) upon reasonable request.
